# Alcohol, Self-Regulation and Partner Physical Aggression: Actor-Partner Effects Over a Three-Year Time Frame

**DOI:** 10.3389/fnbeh.2018.00130

**Published:** 2018-07-05

**Authors:** Brian M. Quigley, Ash Levitt, Jaye L. Derrick, Maria Testa, Rebecca J. Houston, Kenneth E. Leonard

**Affiliations:** ^1^Department of Medicine, Jacobs School of Medicine and Biomedical Sciences, University at Buffalo, Buffalo, NY, United States; ^2^Research Institute on Addictions, University at Buffalo, The State University of New York, Buffalo, NY, United States; ^3^Department of Psychology, University of Houston, Houston, TX, United States; ^4^Department of Psychology, Rochester Institute of Technology, Rochester, NY, United States

**Keywords:** alcohol, self-regulation, partner aggression, executive functioning, self-control

## Abstract

The question of how individual differences related to self-regulation interact with alcohol use patterns to predict intimate partner aggression (IPA) is examined. We hypothesized that excessive drinking will be related to partner aggression among those who have low self-regulation. In addition, we explored the extent to which differences in self-regulation in one partner may moderate the relationship between alcohol use and partner aggression. A sample of married or cohabitating community couples (*N* = 280) ages 18–45 was recruited according to their classification into four drinking groups: heavy drinking in both partners (*n* = 79), husband only (*n* = 80), wife only (*n* = 41), by neither (*n* = 80), and interviewed annually for 3 years. IPA, drinking, and scores on measures of negative affect, self-control, and Executive Cognitive Functioning (ECF) were assessed for both members of the couple. The Actor Partner Interdependence Model (APIM) was used to analyze longitudinal models predicting the occurrence of IPA from baseline alcohol use, negative affect, self-control and ECF. Actor self-control interacted with partner self-control such that IPA was most probable when both were low in self-control. Contrary to prediction, actors high in alcohol use and also high on self-control were more likely to engage in IPA. Partner alcohol use was predictive of actor IPA when the partner was also high in negative affect. Low partner ECF was associated with more actor IPA. These findings suggest that self-regulatory factors within both members of a couple can interact with alcohol use patterns to increase the risk for relationship aggression.

## Introduction

One of the most consistent predictors of intimate partner aggression (IPA) is excessive use of alcohol. Numerous cross-sectional and prospective studies have shown that excessive drinking, particularly male drinking, is associated with the occurrence and frequency of partner violence (see Foran and O’Leary, 2008 for a review). Despite the consistent association, few believe that excessive drinking exerts either a necessary or sufficient condition for partner aggression, but rather that it contributes or facilitates the occurrence of aggression. Moreover, there is recognition that any influence of alcohol on partner aggression is conditional, moderated by both situational and individual difference factors. From the beginning, theoretical approaches to understanding the role of excessive drinking on aggression have sought to address the fact that excessive drinking does not lead to aggression in all people or under all circumstances. Taylor and Leonard (1983) argued that alcohol’s cognitive disruption “might facilitate aggressive behavior in the presence of dominant, instigative cues by increasing one’s attention to those cues and … reducing one’s attention to incompatible, inhibitory cues” (p. 96). This position was expanded upon and formalized as Alcohol Myopia Theory by Steele and Josephs (1990), currently the predominant model of intoxicated behavior. The multiple thresholds model of alcohol-related aggression (Fals-Stewart et al., 2005; Leonard and Quigley, 2017) argued that the impact of alcohol depends on the balance and salience of instigatory and inhibitory cues. Similar to the I^3^ model of aggression (Finkel, 2014), these theories all suggest that alcohol is most likely to facilitate aggression among individuals and in situations characterized by high instigation or low inhibition.

Experimental data generally supports the perspective that people with chronically low self-regulatory abilities become aggressive more easily when drinking. Bailey and Taylor (1991) found that alcohol facilitated aggressive behavior among men who were moderate to high in hostility (an instigatory factor), but not among men low in hostility. Alcohol was more likely to increase aggression for people with high levels of trait anger and irritability as compared to low levels (Giancola, 2002a,b, 2004; Parrott and Zeichner, 2002). Similarly, several studies have suggested that executive functioning is an important moderator of the alcohol aggression relationship. Pihl et al. (2003) reported that individuals with low scores on Executive Cognitive Functioning (ECF) were more aggressive than other groups when administered alcohol, but only under low provocation. Giancola (2004) observed that alcohol increased aggression for men who were low on ECF under both low and high provocation.

Studies of IPA have similarly suggested that the alcohol-aggression relationship is moderated by the influence of both impelling and inhibiting factors in aggression. Heavy drinking is associated with marital violence only among hostile (Leonard and Blane, 1992) or maritally distressed couples (Leonard and Senchak, 1993; Margolin et al., 1998). Quigley and Leonard (1999) demonstrated that heavy alcohol involvement predicted subsequent aggression only among couples high in verbally aggressive conflict styles. Schumacher et al. (2008) found that excessive drinking by the husband longitudinally predicted IPA in men high on hostility and avoidance coping. Based on these findings, Schumacher et al. (2008) asserted “alcohol should have a more pronounced effect on individuals with aggressive perceptual and behavioral propensities, and among individuals, who for dispositional or situational reasons, already have some degree of impaired behavioral regulation and control” (p. 895).

Because aggression is an interactional phenomenon (Tedeschi and Felson, 1994), it is important to examine impelling and inhibitory factors associated with both husbands and wives to fully understand the relationship between alcohol and aggression in couples. Testa et al. (2012) found that husband heavy drinking was associated with violence regardless of wife’s drinking; however at lower levels of husband drinking, heavier drinking by the wife predicted husband aggression. Husband and wife instigating and inhibiting behaviors may exacerbate or ameliorate conflicts in the relationship. In a study using daily diary methods, the presence of an instigator (e.g., provocation) on a given day was most likely to lead to IPA when the partner was high on dispositional aggression and low on a measure of ECF (Finkel et al., 2012). Similarly, both partner’s lack of inhibitory factors could increase the likelihood of aggression. Individuals involved in a relationship have been found to benefit when both are high in self-control but to experience more problems when neither partner has high self-control (Vohs et al., 2011; Crane et al., 2014; Derrick et al., 2016). Thus, while conflict is present in all relationships, impelling and inhibitory factors may influence the course of that conflict and moderate the impact of excessive drinking on aggression.

**Figure 1:**
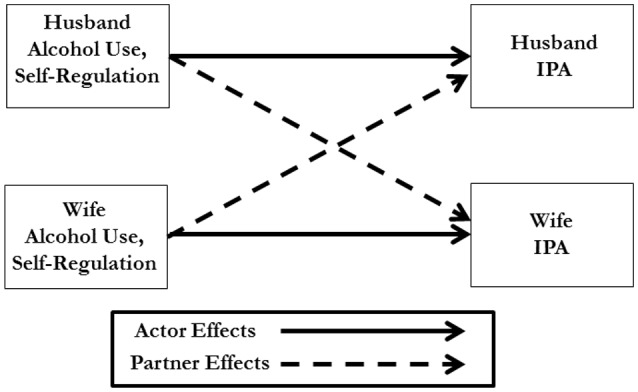
Model of the Actor Partner Interdependence Model (APIM) as used for the present analysis. In APIM both individuals are nested within couple allowing for the simultaneous estimation of effects of each members’ characteristics on their own behavior and the other member of the couple’s behavior. This allows estimation of actor effects and partner effects on behavior independent of gender.

In a previous article (Testa et al., 2012), we examined how the interaction between husband and wife alcohol dependence symptoms predicted intimate partner violence in a cross-sectional analysis of the current data set at the first measurement point in this study. In the present article, we extend that analysis to examine how husband and wife individual differences in self-regulation may interact with alcohol use to predict partner aggression. Because we wished to examine how individual differences in each partner interacted with their own alcohol use and with the alcohol use of the partner to predict IPA by either member of the couple we chose to analyze the data using the Actor Partner Interdependence Model (APIM; Kenny et al., 2006). In a traditional regression analysis framework, it would have been necessary to conduct separate analyses for each member of the couple, however, in an APIM analysis both husband and wife data are nested within the couple. As is shown in Figure 1, which presents the basic APIM model, a dyadic analysis allows the estimation of actor effects and partner effects for each member of the couple. Because observations of each member of the dyad are considered interdependent and nested within couple, we are able to examine the effects of the actor’s drinking and self-control, the effects of the partner’s drinking and self-control, and the effects of any interactions between actor and partner variables on IPA by each member of the couple simultaneously.

The examination of multiple measures of self-control, both self-report and behavioral measures, provide a unique opportunity to examine how differing aspects of self-regulation may interact with alcohol to predict IPV. Based on our earlier work (Schumacher et al., 2008; Testa et al., 2012; Leonard et al., 2014), we hypothesize that excessive drinking will be related to partner aggression among those who have self-regulatory challenges. In addition, we explore the extent to which self-regulation factors in one partner may moderate the relationship between heavy alcohol use and aggressive behavior in the other partner. We examine these hypothesizes over a three-year time period. Based on past research (see Quigley and Leonard, 1999), we expected IPA to decrease over time, however, we also hypothesized that the proposed interactions between drinking and self-regulation will predict IPA both cross-sectionally and longitudinally.

## Materials and Methods

### Participants

A sample of married or cohabitating couples (*N* = 280) was recruited from the community via a mail survey of health behaviors in Erie County, NY, USA. A list of households in Erie County, NY, USA likely to contain households with a head of household between the ages of 18 and 45 was purchased from Survey Sampling International. From this purchased list, 21,000 screening questionnaires were mailed to households accompanied by a letter explaining the purpose of the study. The letter stated that the purpose of the study was to estimate the number of different types of families and to determine the eligibility of respondents and their interest in participating in one of the ongoing studies on families and health. We enclosed a non-contingent dollar bill incentive in the questionnaire to improve response rates (see Homish and Leonard, 2009) and provided a stamped envelope to return the questionnaire. We received 5463 responses for a 26% response rate (226 or about 1% were returned due to an incorrect address). Of the 5463 responses, 10.7% were minorities, with 7.6% being African-American, similar to census data for married couples in Erie County.

Responses from the mailed questionnaire were used to assess study eligibility (between the ages of 18 and 45, and married or living together for at least 1 year) and to determine husband and wife heavy episodic drinking (HED) status. Because one aim of the study involved ECF, we excluded couples if either member had a current medical condition that would impair ECF or if either reported having had a seizure, epilepsy, or a 10-min loss of consciousness due to an accident or head injury. In order to ensure adequate numbers of heavy drinking husbands and wives, we utilized disproportionate sampling to recruit couples in which either member of the couple engaged in regular HED. HED was defined as becoming intoxicated or having five or more drinks at one time (four drinks for women) at least weekly. Our goal was to recruit 75 couples in each of four groups; (1) husband and wife both engaged in HED (Both); (2) only the husband engaged in HED (Husband Only); (3) only the wife engaged in HED (Wife Only); and (4) neither engaged in HED (Control).

Of the 5463 responses, 3477 met eligibility criteria. Of those meeting eligibility criteria, three quarters (75%) of the couples were classified as Control. The rates for Husband Only, Wife Only, and Both were 12.3%, 4.1% and 8.5%, respectively. We also asked whether the couple was interested in participating in one of our ongoing studies. Across the four groups, 68% (*N* = 2347) were interested in participating or hearing more about the studies. The proportion of those who were interested was significantly higher for Husband Only (72%), Wife Only (74%), and Both (76%) than for Control (67%; χ^2^_(3)_ = 16.32, *p* < 0.01). We sampled from the four groups at different rates in order to achieve the goal of 75 couples in each of the four groups. This disproportionate sampling was by design and has implications for our data analyses. We were able to recruit 80 Control, 80 Husband Only, 79 Both, and 41 Wife Only couples. This was a 43% success rate from those who we attempted to recruit, a rate that did not differ across the four groups (χ^2^_(3)_ = 2.78, *p* > 0.40). This indicates that the difficulty that we experienced filling the Wife Only cell reflected the rarity of this group in the population, and not any difference in willingness to participate among these couples.

The average age of the final sample at Time 1 was similar between husbands and wives (36.9, *SD* = 5.8; 35.4, *SD* = 5.9) respectively. The majority of men and women in the sample were White (91% each), highly-educated (58% of husbands and 67% of wives had completed college education compared to 39% for the county), and most were employed at least part-time (91% of husbands and 80% of wives). The majority of couples were married (87%) as opposed to cohabiting and had been together for an average of 9.84 years (*SD* = 5.41). Approximately 79% had children. Among those with children, 15% had one child, 38% had two, 19% had three, and 7.5% had four or more. Median income for wives was in the $20,000–29,999 range, and median income for husbands was in the $40,000–54,999 range.

Out of the 280 couples who participated at Wave 1, 259 (92.5%) completed the assessment at the 1 year anniversary (Wave 2). At the second anniversary (Wave 3), 243 couples completed the assessment (87% of original sample). The present analysis utilizes statistical procedures (PROC Glimmix in SAS 9.4) that allow for the use of all available data at each time point rather than deleting data listwise if the second or third time point is missing.

### Procedure

Participants completed a series of questionnaires sent and returned through the mail and subsequently attended a laboratory assessment. Both members of the couple provided written informed consent to participate in the research at the time of the mail assessment and again at the time of the laboratory assessment. Mailed questionnaires, sent separately to husband and wife, consisted of background information, attitudes and beliefs about alcohol, and personality measures. Participants were instructed to complete questionnaires independently and not to discuss the questionnaires until both had been returned. At the laboratory assessment, partners independently completed computerized questionnaires that addressed relationship issues and alcohol and drug use. In addition, we administered measures of ECF and conducted a semi-structured face-to-face interview regarding one or more episodes of marital conflict. Participants were assured of confidentiality and that their responses would not be shared with their partners. At the first and second anniversary couples again completed both assessments.

### Measures

#### Demographics

Information collected from each partner included age, race, years married and/or years living together, number of children living in the home, and education. Partner reports of age (*r* = 0.83), total years living together (*r* = 0.96) and number of children living in the home (*r* = 0.97) were highly correlated.

#### Intimate Partner Aggression (IPA)

Husband and wife perpetrated IPA over the past 12 months was assessed using the physical aggression subscales of Revised Conflict Tactics Scales (CTS-2, Straus et al., 1996). Each partner reported on the frequency of 12 aggressive acts perpetrated by the self (e.g., “I slapped my partner”) and the same 12 acts as perpetrated by the partner (“my partner slapped me”). Frequency of each act was recorded using the following scale: never (0), once (1), twice (2), 3–5 times (3), 6–10 times (4), 11–20 times (5) and more than 20 times (6). The present analysis examined only the presence or absence of IPA. If either partner reported any act of aggression by the husband toward the wife it was considered IPA by the husband and if either partner reported an act of aggression by the wife toward the husband it was considered an occurrence of IPA by the wife. Thus, in the APIM analysis, each individual nested within couple had their own unique IPA score (1 or 0).

#### Alcohol Dependence

The 25-item Alcohol Dependence Scale (ADS, Skinner and Allen, 1982) was used to assess self-reported occurrence of symptoms of dependence such as blackouts and seeing things that weren’t really there. As expected, the distribution of ADS scores was highly skewed, with 41.9% of men and 55.2% of women reporting scores of 0, but just 4.7% of men and 1.9% of women scoring greater than 9. Scores were Winsorized to reduce the impact of extremely high scores (Reifman and Keyton, 2010).

#### Self-Report Measures of Negative Affect and Self-Control

A general measure of negative affect was assessed with the *Multidimensional Personality Questionnaire* (Tellegen, 1982). The short version of the *Self-Control Scale* (Tangney et al., 2004) was also administered as a self-report inhibitory factor. This measure of a person’s general tendency toward self-control has good internal consistency (α > 0.89) and has been shown to be predictive of aggression in laboratory studies (DeWall et al., 2007).

#### Executive Cognitive Functioning

ECF has often been viewed as an element of cognitive control. Because there is no overall measure of ECF, three types of cognitive tests were used to assess the construct and standardized scores from these tests were combined into a composite ECF score. Past research (Giancola, 2004; Godlaski and Giancola, 2009) has shown alcohol to have stronger effects of aggressive behavior among those low on ECF. During the face to face interview we assessed multiple aspects of ECF assessing cognitive flexibility, attentional selectivity and control, and working memory. These were assessed by the following measures: *Stroop Color-Word Task* (Stroop, 1935; Golden and Freshwater, 2002). The Stroop Task assesses the ability to inhibit an over-learned response and attentional control. Participants were asked to either read words (Word) or name the ink color (Color, Color-Word) for as many stimuli as they could in 45 s. A Color-Word interference score was calculated as outlined in Golden and Freshwater (2002). Lower scores indicate poorer attentional control. *WAIS-III Digit Span* (The Psychological Corporation, 1999). A sequence of digits (one digit per second) was read to the participant. For the Forward condition, the participant was asked to repeat the sequence in the same order. For the Backward condition (working memory), the participant was asked to repeat the sequence in the reverse order. The number of digits increased with successful trials. The number of successful trials were summed for total scores. The Backward score was subjected to a square root transformation. *Trail Making Test* (TMT; Reitan, 1958). The TMT is a measure of cognitive flexibility, visual attention and motor speed (Lezak et al., 2004). In TMT-A, the participant must draw a line connecting a series of numbers in sequential order. TMT-B requires the participant to draw a line connecting a series of letters and numbers alternating between sequential and alphabetical order. Two scores are derived: the completion time (seconds) and number of errors. Completion time scores were subjected to logarithmic transformations while the errors were subjected to inverse transformations.

The measures of ECF, while related to different cognitive functions such as cognitive flexibility, working memory and attention were meant to assess aspects of prefrontal cortex (PFC) functions (Luria, 1980). In order to calculate a composite score of ECF, based on the procedures used by Giancola (2004), who factor analyzed behavioral ECF measures before combining them into single index, we standardized the TMT-B completion time score, the Stroop Interference score, and the Digit Span total score and summed the three scores together.

## Results

### Data Analysis Plan

Multilevel analyses were conducted using the Actor-Partner Interdependence Model (Kenny et al., 2006). Models were estimated using SAS PROC Glimmix (Version 9.4; SAS Institute Inc., 2017), and predicted the probability of physical IPA occurrence. Repeated measures at Level 1 were crossed between partners and nested within couple at Level 2 (Laurenceau and Bolger, 2005; Kashy and Donnellan, 2012), allowing for missing data at Level 1. We took into account the disproportionate sampling of the couple drinking groups by weighting the participants in the different groups to reflect their prevalence among the eligible respondents to our mailed survey. Probability weights were used in all models due to the nature of our sample, which oversampled various combinations of heavy drinking partnerships (Pfeffermann, 1993; Korn and Graubard, 1995). The outcome variable, IPA occurrence, was treated as time varying across the three waves of data. Predictor variables were taken from the baseline assessment only and were therefore time-invariant. Time was centered at baseline and increased yearly across the three waves of data. Linear growth trajectories of IPA occurrence over the three study waves were estimated. Main effects of predictors indicate intercept differences in IPA occurrence, and interactions between predictors and time indicate differences in the linear growth of IPA occurrence. All models allowed for random intercept and error components, and all predictors were grand mean centered and entered as fixed effects.

Preliminary models were run to test the effects of potential baseline covariates including age, children (yes/no), years living together, and education of each partner on the occurrence of IPA. No covariates were significant predictors of IPA in the presence of any of the predictors of interest and were not included in the final models. Preliminary models were also run to test whether men and women were empirically distinguishable in the means, variances, and covariances of the current set of variables (Kenny et al., 2006; Kashy and Donnellan, 2012). Models including gender effects fit significantly worse than indistinguishable models for all three predictors of interest: negative affect (χ^2^diff = −34.04, *p* < 0.001), self-control (χ^2^diff = −20.80, *p* = 0.023), and ECF (χ^2^diff = −77.06, *p* < 0.001). Therefore, models were treated as indistinguishable and gender differences were not tested.

### Rates of Partner Physical Aggression Perpetration and Alcohol Dependence

Because this was a general population sample, the rates of violence were not at the levels of those from a clinical sample but were still frequent enough to allow analysis. At Wave 1, 31.8% of the women and 22.1% of the men perpetrated at least one act of physical aggression based on the maximum reports of both partners. Rates fell over the next two time periods. At Wave 2, 23.3% of the women and 17.0% of the men engaged in at least one act of physical aggression against their partner and at Wave 3 these rates reduced to 21.0% and 12.8% respectively. Although we did not assess treatment seeking for alcohol use or domestic violence, the reduction in IPA over time is consistent with past research on non-treatment seeking samples (Quigley and Leonard, 1996). Alcohol dependence scores stayed relatively stable for men over the 3-year time frame changing from average scores of 3.99 (*sd* = 4.55) at Wave 1 to 3.88 (*sd* = 4.43) at Wave 2 and 3.83 (*sd* = 4.83) at Wave 3 for men and average scores of 2.88 (*sd* = 4.10) at Wave 1 to 2.58 (*sd* = 3.76) at Wave 2 and 2.36 (*sd* = 3.88) at Wave 3 for women.

### APIM Models Predicting Partner Aggression

#### Negative Affect

**Figure 2:**
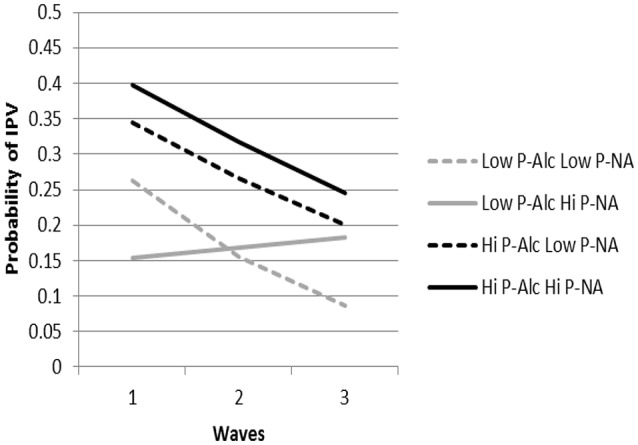
The predicted probabilities of IPA for P-Alc by P-NA at each time point are presented. Partners high on negative affect and alcohol use showed the highest initial probability of actor IPA. Partners high in alcohol use and low on negative affect and partners low on alcohol use and high on negative showed the next highest probability of actor IPA. These groups all reduced their IPA over time while partners low in alcohol use but high on negative affect had the lowest predicted probabilities of actor IPA at the first time point and maintained the same level over the three time points. P-Alc = Partner Alcohol; P-NA = Partner Negative Affect.

We first examined growth trajectories of actor IPA occurrence as a function of actor and partner alcohol dependence symptoms and negative affect (χ^2^_(273)_ = 651.66, *p* < 0.001). There were main effects of both actor and partner alcohol use indicating greater alcohol use was associated with more IPA (see Table 1). There was also a partner alcohol by actor alcohol interaction effect, however, as the associated cross-sectional effect was discussed in Testa et al. (2012) we won’t discuss it further here. Both actor and partner negative affect interacted with time indicating that those high and low in negative affect diverged in IPV over time with greater negative affect being associated with greater odds of IPA. There was a significant interaction between partner alcohol use and partner negative affect. Although the three-way interaction effect of partner alcohol use by partner negative affect by time did not reach the traditional level of statistical significance (*p* = 0.051) we find it useful to interpret the significant two-way interaction in light of the moderating effect of time. As shown in Figure 2, high partner alcohol dependence predicted actor IPA occurrence relative to low partner alcohol dependence, with the highest probability occurring when the partner was also high in negative affect. Both of these trajectories also decreased over the study period. In contrast, the trajectories of IPA occurrence for low partner alcohol use did not uniformly decrease over time as a function of partner negative affect. As expected, physical IPA occurrence as a function of low partner alcohol dependence and low partner negative affect decreased to the lowest levels over time. However, low partner alcohol dependence and high negative affect was the only combination to not decrease in the probability of IPA occurrence over time. Overall, partner negative affect has a differential effect on actor IPA occurrence at low levels of partner alcohol use but not high levels of partner alcohol use.

**Table 1:** Tests of baseline associations between drinking and negative affect on growth trajectories of occurrence of intimate partner violence.

					95% CI	
Predictors	b	(SE)	*t*	*p*	Lower	Upper
**Intercept**	**−0.974**	**(0.151)**	**−6.46**	**<0.001**	**−1.271**	**−0.678**
**Time**	**−0.310**	**(0.129)**	**−2.41**	**0.016**	**−0.563**	**−0.057**
**A-Alcohol**	**0.117**	**(0.033)**	**3.56**	**<0.001**	**0.052**	**0.182**
**P-Alcohol**	**0.123**	**(0.033)**	**3.68**	**<0.001**	**0.057**	**0.188**
A-Negative Affect	0.510	(0.620)	0.82	0.411	−0.706	1.726
P-Negative Affect	−0.661	(0.627)	−1.05	0.292	−1.890	0.569
A-Alcohol * P-Alcohol	**−0.017**	**(0.008)**	**−2.12**	**0.034**	**−0.032**	**−0.001**
A-Negative Affect * P-Negative Affect	−5.208	(3.529)	−1.48	0.140	−12.135	1.719
A-Alcohol * A-Negative Affect	−0.075	(0.157)	−0.48	0.633	−0.384	0.233
A-Alcohol * P-Negative Affect	0.130	(0.169)	0.77	0.440	−0.200	0.461
P-Alcohol * A-Negative Affect	0.137	(0.183)	0.75	0.454	−0.221	0.495
P-Alcohol * P-Negative Affect	**0.354**	**(0.169)**	**2.10**	**0.036**	**0.023**	**0.684**
A-Alcohol * Time	−0.046	(0.029)	−1.57	0.118	−0.103	0.012
P-Alcohol * Time	−0.014	(0.028)	−0.49	0.625	−0.070	0.042
A-Negative Affect * Time	**1.181**	**(0.527)**	**2.24**	**0.025**	**0.147**	**2.215**
P-Negative Affect * Time	**1.119**	**(0.529)**	**2.12**	**0.035**	**0.081**	**2.157**
A-Alcohol * A-Negative Affect * Time	0.021	(0.146)	0.14	0.886	−0.266	0.308
A-Alcohol * P-Negative Affect * Time	−0.093	(0.134)	−0.69	0.488	−0.357	0.170
P-Alcohol * A-Negative Affect * Time	−0.006	(0.140)	−0.04	0.968	−0.280	0.268
P-Alcohol * P-Negative Affect * Time	*−0.293*	*(0.150)*	*−1.95*	*0.051*	*−0.588*	*0.001*

*Note. A, actor; P, partner. Bold items indicate effect significant at p < 0.05. Italic items indicate effect nearing significance at p < 0.10*.

#### Self-Control

**Figure 3:**
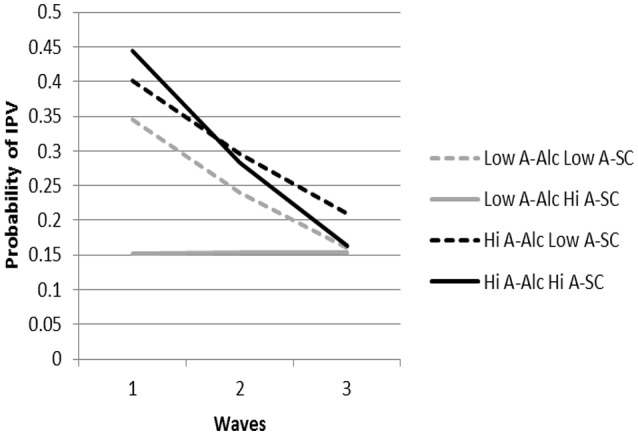
The predicted probabilities of intimate partner aggression (IPA) for A-Alc by A-SC at each time point are presented. Actors high on self-control and alcohol use showed the highest initial probability of IPA. Actors high in alcohol use and low on self-control and actors low on alcohol use and low on self-control showed the next highest probabilities of IPA. These groups all reduced their IPA over time. Actors low on alcohol use but high on self-control had the lowest predicted probabilities of IPA over all time points. A-Alc = Actor Alcohol; A-SC = Actor Self-control.

We next examined growth trajectories of actor IPA occurrence as a function of actor and partner alcohol dependence and self-control (χ^2^_(325)_ = 706.83, *p* < 0.001). As shown in Table 2, there was a significant actor alcohol use by actor self-control by time interaction. As shown in Figure 3, at baseline, all combinations of actor alcohol dependence and actor self-control, with one exception, predicted relatively similar probabilities of actor IPA occurrence, and similarly declined over time. Low actor alcohol dependence and high actor self-control was notably lower than the other combinations at baseline and did not change over time. Overall, differences in alcohol dependence were not apparent at low levels of self-control, whereas there was a marked difference in IPA occurrence at baseline and over time for low vs. high alcohol dependence for those high in self-control.

**Table 2:** Tests of baseline associations between drinking and self control on growth trajectories of occurrence of intimate partner violence.

					95% CI	
Predictors	b	(SE)	*t*	*p*	Lower	Upper
**Intercept**	**−0.761**	**(0.155)**	**−4.92**	**<0.001**	**−1.064**	**−0.457**
**Time**	**−0.412**	**(0.132)**	**−3.12**	**0.002**	**−0.672**	**−0.153**
**A-Alcohol**	**0.122**	**(0.034)**	**3.55**	**<0.001**	**0.055**	**0.189**
**P-Alcohol**	**0.151**	**(0.034)**	**4.42**	**<0.001**	**0.084**	**0.218**
**A-Self-Control**	**−0.422**	**(0.185)**	**−2.28**	**0.023**	**−0.785**	**−0.059**
P- Self-Control	0.056	(0.184)	0.31	0.760	−0.304	0.417
A-Alcohol * P-Alcohol	**−0.023**	**(0.009)**	**−2.61**	**0.009**	**−0.040**	**−0.006**
A-Self-Control * P-Self-Control	**0.813**	**(0.276)**	**2.94**	**0.003**	**0.271**	**1.356**
A-Alcohol * A-Self-Control	**0.156**	**(0.054)**	**2.89**	**0.004**	**0.050**	**0.262**
A-Alcohol * P-Self-Control	−0.033	(0.052)	−0.64	0.520	−0.135	0.068
P-Alcohol * A-Self-Control	0.010	(0.052)	0.19	0.853	−0.093	0.112
P-Alcohol * P-Self-Control	0.076	(0.053)	1.45	0.147	−0.027	0.180
A-Alcohol * Time	−0.046	(0.031)	−1.51	0.131	−0.107	0.014
P-Alcohol * Time	−0.029	(0.030)	−0.97	0.333	−0.087	0.029
A-Self-Control * Time	0.141	(0.156)	0.90	0.369	−0.166	0.447
P-Self-Control * Time	0.007	(0.155)	0.05	0.963	−0.296	0.311
A-Alcohol * A-Self-Control * Time	**−0.094**	**(0.045)**	**−2.11**	**0.035**	**−0.182**	**−0.007**
A-Alcohol * P-Self-Control * Time	0.031	(0.039)	0.81	0.416	−0.044	0.107
P-Alcohol * A-Self-Control * Time	−0.009	(0.039)	−0.24	0.808	−0.085	0.066
P-Alcohol * P-Self-Control * Time	−0.037	(0.044)	−0.83	0.406	−0.123	0.050

*Note. A, actor; P, partner. Bold items indicate effect significant at p < 0.05. Italic items indicate effect nearing significance at p < 0.10*.

**Figure 4:**
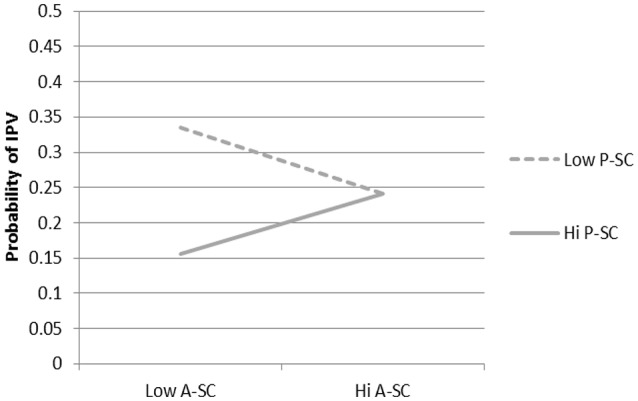
Predicted Probabilities of IPA for A-SC by P-SC. Among actors with high self-control the partner’s self-control was not predictive of the probability of actor IPA. However, among actors low on self-control, low partner self-control was associated with a greater probability of actor IPA than when the partner was high on self-control. A-SC = Actor Self-control; P-SC = Partner Self-control.

There was also a significant actor by partner self-control interaction (see Figure 4). Among those with a partner low in self-control, the highest probability of actor IPA perpetration was when the actor was also low in self-control, whereas the lowest probability of actor IPA occurrence was when the actor was low in self-control and the partner was high in self-control. Probabilities of actor IPA occurrence did not differ as a function of partner self-control when the actor was high in self-control and fell between the two trajectories previously described. This interaction was not moderated by time.

#### Executive Cognitive Functioning

We examined growth trajectories of actor IPA occurrence as a function of alcohol use and ECF (χ^2^_(295)_ = 656.31, *p* < 0.001). There were no moderating effects of time on alcohol use and ECF interactions (i.e., three-way interactions) so time was dropped from these analyses. There was a main effect of partner ECF indicating greater actor IPA when the partner was low on ECF (Table 3). Theory would have predicted a significant interaction between actor alcohol use and actor ECF predicting actor IPA occurrence, however, in this sample that interaction did not reach statistical significance (*p* = 0.062). Because of the importance of that interaction to the threshold model and the fact that the choice of an alpha level, while a common convention, is somewhat arbitrary we elected to tentatively examine the shape of that interaction. As would be predicted, the highest probability of actor IPA perpetration was found for actors high in alcohol use but low in ECF. No effect of ECF was found among those low in alcohol use. While this finding is consistent with theory, because the effect did not reach the traditional level of statistical significance we suggest caution in interpretation of this effect.

**Table 3:** Tests of baseline associations between drinking and executive cognitive functioning (ECF) on occurrence of intimate partner violence.

					95% CI	
Predictors	b	(SE)	*t*	*p*	Lower	Upper
**Intercept**	**−0.904**	**(0.130)**	**−6.98**	**<0.001**	**−1.158**	**−0.650**
**Time**	**−0.294**	**(0.106)**	**−2.77**	**0.006**	**−0.503**	**−0.086**
**A-Alcohol**	**0.088**	**(0.020)**	**4.47**	**<0.001**	**0.049**	**0.126**
**P-Alcohol**	**0.116**	**(0.020)**	**5.88**	**<0.001**	**0.077**	**0.154**
A-ECF	−0.055	(0.035)	−1.58	0.115	−0.123	0.013
**P-ECF**	**−0.073**	**(0.035)**	**−2.10**	**0.036**	**−0.141**	**−0.005**
A-Alcohol * P-Alcohol	*−0.013*	*(0.007)*	*−1.85*	*0.065*	*−0.028*	*0.001*
A-ECF * P-ECF	0.001	(0.017)	0.05	0.959	−0.033	0.034
A-Alcohol * A-ECF	*−0.021*	*(0.011)*	*−1.87*	*0.062*	*−0.042*	*0.001*
A-Alcohol * P-ECF	0.011	(0.010)	1.08	0.281	−0.009	0.030
P-Alcohol * A-ECF	0.009	(0.010)	0.94	0.348	−0.010	0.029
P-Alcohol * P-ECF	−0.017	(0.011)	−1.62	0.106	−0.039	0.004

*Note. A, actor; P, partner. Bold items indicate effect significant at p < 0.05. Italic items indicate effect nearing significance at p < 0.10*.

## Discussion

The findings of the current study suggest that a pattern of excessive drinking is more strongly associated with IPA when moderated by factors related to self-regulation. Use of the APIM framework to analyze the data allowed us to examine how both partners’ characteristics play a role in relationship aggression. It is clear that IPA by one member of a couple is not only a function of that person’s alcohol use and ability to self-regulate but also a function of these same factors within their partner. The findings of the present study were generally consistent with past research examining the moderators of alcohol-related aggression but also help to extend our knowledge regarding the relationship of alcohol use to anger and aggression.

While self-regulation is a broad term that encompasses many constructs, we chose to focus on three aspects that have been found to be related to aggression in past research: ECF, negative affect, and self-control. We examined a number of constructs related to self-control because self-control is a complex behavior involving numerous neurocognitive mechanisms. Past research has suggested that at minimum self-control requires at least two stages that involve different brain centers. When encountering a conflict situation in which self-control may be necessary, recognition of the conflict is usually associated with activity in the anterior cingulate cortex (Kerns et al., 2004). Actual impulse control however, seems mostly controlled within the domain of the prefrontal cortex, in particular areas such as the dorsolateral prefrontal cortex (MacDonald et al., 2000) and the ventrolateral prefrontal cortex (Tabibnia et al., 2011).

Actor self-report of self-control interacted with actor alcohol use to predict the occurrence of aggression, however, actors *high* in levels of self-control had a greatest probability of IPA when they also were heavy drinkers. Other combinations of alcohol use and self-control were moderately associated with the probability of IPA. The reason for this unexpected finding is not clear, however, Imhoff et al. (2014) found a similar interaction between self-control depletion and an individual difference measure of self-control predicting eating restraint and task persistence. In two studies, individuals scoring highly on a self-report measure of self-control similar to the one used here and who had their self-control experimentally depleted showed lower levels of subsequent self-control than those low in self-control who had also been depleted. It was suggested by Imhoff et al. (2014) that individuals scoring high on such a self-report measure of self-control are actually good at *avoiding* situations in which they must exert self-control. Thus, they often have little experience in dealing with situations in which their self-control is put to a test. When their self-control resources are reduced (either by depletion as in the Imhoff studies or by alcohol use as in our study) and they are put in a situation where they must self-regulate, they are actually very poor at self-regulation due to lack of experience. While this is one possible explanation for our finding there are others as well. An examination of the items in this scale suggests that they may be indicative of a controlling or authoritarian personality for some individuals. Recent research with a population of incarcerated batterers demonstrated that batterers were not more impulsive than other criminals but were more cognitively inflexible (Bueso-Izquierdo et al., 2016). Although the levels of violence in the current sample are low, this finding may be indicative of some of the mechanisms that are functioning at more extreme levels in highly violent couples and in cases of intimate terrorism (Johnson, 2008). The findings suggest that this measure may contain a number of artifacts affecting its validity among certain populations. However, that is an empirical question that should be addressed in future research.

The predicted interaction of actor alcohol use with actor ECF failed to reach the traditional level of statistical significance, however, the form of the interaction suggested that actors who were heavy drinkers and low on ECF may be more likely to commit IPA. Past research has demonstrated that individuals seeking alcohol abuse treatment who have a history of partner violence show more cognitive deficits in attention, concertation and cognitive flexibility than similar treatment seeking men without a history of partner violence (Easton et al., 2008) and laboratory studies have shown that individuals low on ECF are more aggressive when intoxicated (Giancola, 2004; Godleski and Giancola, 2009). While other research has found effects of certain aspects of ECF such as impulsivity (Hoaken et al., 2003; Schumacher et al., 2013) we used a composite score of ECF similar to that used by Giancola (2004) which combined measures of cognitive flexibility, attentional control, and working memory in order to provide a wide-ranging assessment of prefrontal cortex functioning. Previous research using similar ECF measures to ours which did find this interaction were highly controlled laboratory studies, suggesting that our inability to find a significant effect may be due to the significant amount of uncontrolled variability that is endemic of non-experimental research. Although we should interpret our finding with caution due to the failure to reach traditional levels of statistical significance, the pattern is consistent with the theory that heavy alcohol use when combined with a poor self-regulation may be a dangerous combination in the context of a relationship.

The data was analyzed in an APIM framework in order to examine partner and actor effects simultaneously. Two recent studies show showed that deficits in actor self-regulation predicted IPA and that partner alcohol use diminished the effect of actor self-regulation on aggression. In a study by Leone et al. (2016), an aspect of actor impulsiveness (negative urgency) predicted IPA among those whose partners were not heavy drinkers, however, this relationship between actor impulsiveness and IPA was attenuated when the partner was a heavy drinker. Having a heavy drinking partner was associated with actor IPA regardless of the actor’s self-regulation. Similarly, Parrott et al. (2017) fund that the relationship between actor emotional regulation and IPA was stronger when the partner was not a problem drinker than when the partner was a problem drinker. Again, partner problem drinking had the effect of predicting IPA but it also reduced the relationship between self-regulation and IPA. We didn’t find actor self-regulation by partner alcohol use interactions, but we did find interactions involving partner effects.

First, partner alcohol use interacted with partner negative affect to predict the probability of actor IPA suggesting that actors are more likely to be aggressive when a partner is high on negative affect and also heavy drinker. There are several possible explanations for this finding. One may be that the partner is also more likely to be aggressive and the members of the couple engage in reciprocal aggression. However, the lack of an actor negative affect by actor alcohol use interactions suggests that this is too simple an explanation. It may rather be indicative of a high conflict relationship. Past research has found couple conflict to interact with alcohol use to predict partner aggression (e.g., Quigley and Leonard, 1999). A partner who is frequently angry and intoxicated may more often be responded to with physicality regardless of the characteristics of the actor. The second interaction involving partner effects also suggests that greater conflict leads to more relationship aggression. Actor self-control interacted with partner self-control such that IPA was most probable when both actor and partner were low in self-control and least probable when the actor was low in self-control and the partner was high in self-control. When the actor was high in self-control the partner’s self-control did not matter. While it has been established that individuals low in self-control are more likely to enact IPA (Finkel et al., 2009) and that low self-control is a risk factor for experiencing victimization (Pratt et al., 2014) this is the first research to show that both partner’s self-control interacts to predict IPA.

The interaction found with time did not suggest that the relationship of self-regulation and alcohol use to IPA changes over time, rather it indicated overall reductions in aggression over time. In the three-way interaction involving time, the patterns suggested that the interaction was due to one condition in which the probability of IPA was low at wave one which stayed low over the next two waves while the probability of IPA in other conditions reduced over time. Partner aggression is known to reduce and even desist over time (Quigley and Leonard, 1996; Walker et al., 2013) and this fact is likely partly responsible for the reduction in IPA over time in this sample.

There are a number of strengths to the present analysis. The sample is unique in a number of ways that help address limitations in past research on alcohol and IPA. First, the sample is older than the college age or newlywed samples that are usually examined in IPA research. Secondly, we oversampled heavy drinking women so that we could properly address the understudied issue of heavy alcohol use by women as a risk factor for their own IPA as well as for being victims of IPA. The oversampling of heavy drinking women may be why the APIM models were indistinguishable by gender as past samples rarely had equivalent numbers of heavy drinking men and women. Our findings were consistent with the multiple thresholds model of alcohol-related aggression and indicated that the instigating effects of poor self-regulation in combination with the disinhibiting effects of alcohol creates a situation ripe for relationship aggression. This model can help us to understand how alcohol use and factors related to cognitive, emotional and behavioral control in both individuals in the relationship can influence the occurrence of partner violence. Future research should expand the types of self-regulatory factors examined in order to provide more complete understanding of how basic neurocognitive substrates related to self-control direct and moderate intoxicated behavior. Finally, our findings demonstrate the importance of considering the interactional qualities of marital conflict. While we often simply look at characteristics of the perpetrator, situations of partner aggression involve the interaction of two individuals each with their own patterns of alcohol use, personality differences and levels of cognitive functioning.

## Ethics Statement

The research reported in this manuscript was reviewed and approved by the University at Buffalo’s Social and Behavioral Sciences Institutional Review Board.

## Author Contributions

KL, BQ, MT and RH contributed to the design of the study. BQ, AL and JD contributed to data analysis. All authors contributed to the draft of the manuscript.

## Conflict of Interest Statement

The authors declare that the research was conducted in the absence of any commercial or financial relationships that could be construed as a potential conflict of interest.
